# Identification of core genes mediating the association between obesity and hepatocellular carcinoma: A bioinformatics study based on mitochondrial metabolism and immune pathways

**DOI:** 10.1371/journal.pone.0344452

**Published:** 2026-03-09

**Authors:** Xiaocan Li, Rui Min

**Affiliations:** Joint Program of Nanchang University and Queen Mary University of London, Medical College of Nanchang University, Nanchang, Jiangxi, China; Hokkaido University: Hokkaido Daigaku, JAPAN

## Abstract

**Purpose:**

Obesity is strongly associated with hepatocellular carcinoma (HCC), yet the molecular mechanisms linking them remain unclear. This study aimed to identify mitochondrial metabolism-related genes bridging obesity and HCC and to investigate their role in regulating the metabolic-immune microenvironment.

**Methods:**

Public transcriptomic datasets from obesity (derived from peripheral blood mononuclear cells) and HCC (derived from liver tissue) cohorts were integrated. A multi-step bioinformatic pipeline combining differential expression analysis (DEA), weighted gene co-expression network analysis (WGCNA), and machine learning (ML) algorithms was applied to identify and validate hub genes. Associations with the tumor immune microenvironment were assessed using ssGSEA and correlation analyses.

**Results:**

27 core genes were identified, significantly enriched in lipid metabolism and immune response pathways. Among these, ML highlighted *ACAA1* and *ADI1* as downregulated candidate genes. While discovery datasets showed high diagnostic potential, *ADI1* exhibited more variable performance in obesity external validation compared to the robust consistency of *ACAA1*. Downregulation of both genes correlated with effector T/NK cell lipid-mediated functional exhaustion and disrupted networks of immune checkpoints and chemokines, reflecting an immunosuppressive microenvironment.

**Conclusions:**

*ACAA1* and potentially *ADI1* are downregulated candidate genes linking obesity to HCC. Their suppression likely drives obesity-related HCC progression by coupling mitochondrial metabolic reprogramming with immunosuppressive tumor microenvironment remodeling, representing potential therapeutic targets.

## 1. Introduction

The global rise in overweight and obesity poses a severe public health threat, affecting nearly 2.5 billion adults in 2022. While Western standards define obesity as a BMI > 30, Asian populations, including the Korean cohort analyzed in this study, use a lower threshold of BMI ≥ 25 due to higher metabolic risks at lower BMI levels [1–3]. Beyond excess weight, obesity involves metabolic disturbances like insulin resistance, metabolic dysfunction-associated fatty liver disease (MAFLD), and chronic low-grade inflammation [4–6]. Impaired mitochondrial transfer between adipocytes and macrophages also contributes to metabolic dysregulation [7]. These abnormalities significantly increase the risk of type 2 diabetes mellitus, cardiovascular disease, and several cancers, including breast, colorectal, liver, and pancreatic cancers [8,9].

Hepatocellular carcinoma (HCC), the primary form of liver cancer arising from hepatocytes, ranks as the third leading cause of cancer-related mortality [10–12]. Chronic HBV/HCV infection [13,14], alcohol consumption, and aflatoxin exposure [15] remain major etiological factors, but epidemiological evidence identifies obesity as an independent and increasingly important HCC risk factor. Several Asian studies report that individuals with a BMI ≥ 25 have a 1.5-2-fold higher risk of HCC than their normal-weight counterparts [16]. Obesity promotes the pathological cascade from MAFLD to metabolic dysfunction-associated steatohepatitis (MASH), advancing to cirrhosis and ultimately HCC, thereby markedly elevating cancer risk [17,18]. Mechanistically, dysregulated adipokine secretion (e.g., leptin, adiponectin) combined with increased hepatic endotoxin influx due to impaired intestinal barrier function activates multiple oncogenic pathways in hepatocytes [19,20], including aberrant lipid metabolism and persistent inflammatory signaling [21,22]. The underlying mechanisms are highly complex. Metabolic reprogramming and immune dysregulation likely are pivotal in HCC progression [23].

Mitochondria, central hubs of cellular energy metabolism and signaling, are abundant in the liver and essential for hepatic homeostasis. They serve as the primary site of lipid metabolism, and mitochondrial dysfunction contributes to hepatocellular lipid accumulation in MAFLD [24]. Furthermore, mitochondria-centered metabolic reprogramming is a critical determinant of immune cell differentiation and functional phenotypes within the tumor microenvironment (TME) [25,26]. Thus, mitochondrial dysregulation may exacerbate lipid metabolic abnormalities and immune imbalance, potentially linking obesity to HCC. Nevertheless, current research in this area remains limited, and it is a biological entry point for elucidating intersecting molecular pathways.

Although the association between obesity and HCC has been extensively studied, few investigations have systematically explored key regulatory factors from an integrated metabolic-immune perspective. Mitochondrial metabolism, with its dual role in lipid homeostasis and immune regulation, emerges as a candidate focus. This study integrates transcriptomic datasets of obesity and HCC from GEO and TCGA, employing differential expression analysis (DEA), weighted gene co-expression network analysis (WGCNA), and multiple machine learning (ML) algorithms to identify mitochondrial metabolism-related hub genes. Their associations with immune infiltration are further analyzed to delineate potential molecular cross-talk between obesity and HCC, providing a theoretical foundation for targeted interventions.

## 2. Materials and methods

### 2.1. Data collection and preprocessing

Obesity-related datasets were obtained from the GEO database (https://www.ncbi.nlm.nih.gov/geo/), specifically GSE55205 and GSE69039, both derived from peripheral blood mononuclear cells (PBMCs). GSE55205 included 17 obesity and 6 normal samples, while GSE69039 contained 14 obesity and 4 normal samples. HCC data, comprising 50 paired tumor and adjacent normal tissue samples and 374 unpaired tumor samples, were retrieved from the UCSC Xena platform (https://xena.ucsc.edu/). Two independent GEO cohorts were used for external validation: GSE144269 (liver cancer; 70 HCC and 70 adjacent normal samples) and GSE151839 (obesity; 20 obesity and 20 normal samples). Detailed sample characteristics are summarized in S1Table.

Mitochondrial metabolism-related genes (MMGs) were extracted from the GeneCards database (https://www.genecards.org/). Obesity datasets (GSE55205 and GSE69039) were merged, and batch effects were corrected via sva 3.54.0.

### 2.2. DEA

Differentially expressed genes (DEGs) were identified via limma 3.62.2. For the obesity datasets, genes with |logFC| > 0 and adj.P < 0.05 were considered significant [27], while for the HCC dataset, the threshold was set at |log₂FC| > 1 and adj.P < 0.05. Visualization was performed via ggplot2 3.5.1.

### 2.3. Identification of overlapping genes

Intersecting genes among obesity-related DEGs, HCC-related DEGs, and MMGs were identified and visualized via ggvenn 0.1.10.

### 2.4. WGCNA

Based on the TCGA-LIHC and obesity datasets, the top 3,000 and 8,000 most variable genes were selected for WGCNA. Sample clustering based on Euclidean distance was used to detect outliers. Scale-free networks were constructed with soft-thresholding powers selected as the minimum powers achieving a scale-free topology fit (R² > 0.85) while maintaining mean connectivity. Key modules strongly correlated with phenotypes were identified for subsequent analyses.

### 2.5. Functional enrichment analysis

Genes from obesity- and HCC-associated modules identified by WGCNA were intersected with MMGs, and the results were visualized via ggvenn 0.1.10. The union of these intersecting genes and DEGs was defined as hub genes. Functional enrichment analyses of hub genes were conducted using clusterProfiler 4.14.6, including Gene Ontology (GO) [28] and Kyoto Encyclopedia of Genes and Genomes (KEGG) pathway enrichment [29]. An unadjusted P < 0.05 was considered statistically significant to maximize sensitivity for potential biological signals.

### 2.6. ML

Preliminary feature selection used LASSO regression in glmnet 4.1–8, with optimal λ determined by five-fold cross-validation. Genes with non-zero coefficients were retained as candidate features. Redundant features were eliminated using the support vector machine-recursive feature elimination (SVM-RFE) algorithm, with the optimal subset selected based on accuracy curves from five-fold cross-validation. Candidate genes identified by both LASSO and SVM-RFE across obesity and HCC datasets were intersected to define key genes, which were further analyzed via single-sample Gene Set Enrichment Analysis (ssGSEA).

### 2.7. External validation and diagnostic performance evaluation

The expression of core genes in different groups of obesity and HCC datasets was visualized through boxplots generated by ggstatsplot 0.13.0. Receiver operating characteristic (ROC) curves were constructed using pROC 1.18.5, and the area under the curve (AUC) was calculated. Two independent external cohorts, GSE144269 (HCC) and GSE151839 (obesity), were analyzed using the same approach to assess stability and diagnostic performance.

### 2.8. Immune infiltration analysis

Immune infiltration was assessed via ssGSEA in GSVA 1.50.5. Enrichment scores of selected immune cell types were quantified, with differences visualized using violin and boxplots (ggplot2). Pearson correlations between target genes (*ACAA1* and *ADI1*) and immune cell scores were computed with corrplot 0.95 and displayed as heatmaps. Associations with immune checkpoint and chemokine genes were also evaluated. Significance thresholds were set at P < 0.01 for obesity cohorts and P < 0.001 for the HCC cohort to account for variations in sample size.

### 2.9. Statistical analysis

All analyses were performed in R 4.4.3. Data visualization employed ggplot2 3.5.1 and corrplot 0.95. Intergroup comparisons of continuous variables used the Wilcoxon rank-sum test, while correlations were assessed with Pearson analysis. Unless otherwise specified, P < 0.05 indicated statistical significance.

### 2.10. Ethics approval and consent to participate

All data used in this study were obtained from publicly available databases (TCGA and GEO), and further ethical approval and informed consent were not required. The research was conducted in strict accordance with the data usage policies of the respective repositories.

## 3. Results

### 3.1. Identification of DEGs in obesity and HCC

**Fig 1:**
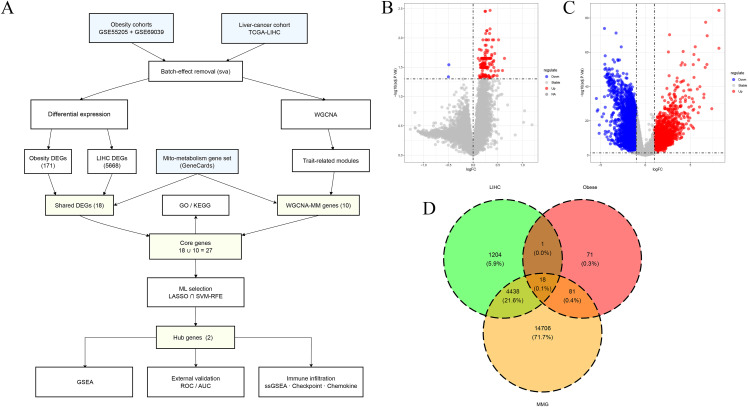
Identification of DEGs. **A.** The flowchart of this study. **B.** Volcano plot of DEGs in the obesity dataset (|logFC| > 0 and adj.P < 0.05). **C.** Volcano plot of DEGs in the HCC dataset (|log₂FC| > 1 and adj.P < 0.05). **D.** Venn diagram illustrating overlapping genes.

The study flowchart is presented in Fig 1A. After batch effect correction (S1 Fig), DEA of the integrated obesity dataset using limma identified 171 DEGs, comprising 169 upregulated and 2 downregulated genes (|logFC| > 0, FDR < 0.05). The volcano plot illustrates the expression patterns between obese and normal groups (Fig 1B). Applying the same pipeline to the TCGA-LIHC dataset yielded 5,661 DEGs, including 1,655 upregulated and 4,006 downregulated genes (|log₂FC| > 1, FDR < 0.05) (Fig 1C). Intersection analysis of DEGs from the obesity and HCC datasets with MMGs highlighted 18 genes significantly dysregulated in both conditions (Fig 1D): *MSTO1*, *CNIH4*, *GNPAT*, *ARPC5*, *POLG2*, *CCT6A*, *KPNA2*, *INTS3*, *NOP56*, *HDAC4*, *ANKRD27*, *INTS8*, *ACAA1*, *OFD1*, *SPRYD4*, *TMOD4*, *HSPA1A*, and *AVIL*.

### 3.2. WGCNA network construction and module identification

**Fig 2:**
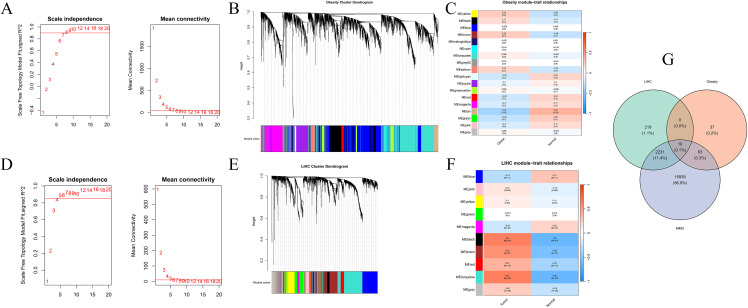
WGCNA of Obesity and HCC Datasets. A. Soft-thresholding power β = 7 for the obesity dataset. B. Dendrogram of co-expressed genes in the obesity dataset; each color represents a distinct module. C. Heatmap of module-trait correlations in the obesity dataset. The color bar indicates the correlation coefficient (r). D. Soft-thresholding power β = 5 for the HCC dataset. E. Dendrogram of co-expressed genes in the HCC dataset; each color represents a distinct module. F. Heatmap of module-trait correlations in the HCC dataset. The color bar indicates the correlation coefficient (r). G. Venn diagram depicting the intersection of significant module genes with MMGs.

WGCNA identified gene modules associated with metabolic shifts. In the obesity dataset, a cut height of 45 was applied based on sample clustering, excluding five outlier samples (S2 Fig). A soft-thresholding power of β = 7 was used to construct the co-expression network (Fig 2A), resulting in 18 modules (Fig 2B). Module-trait correlation analysis indicated that the MEtan module exhibited the strongest association with the obesity phenotype (r = 0.37, p = 0.03) (Fig 2C). Regarding the HCC dataset, a cut height of 130 retained all samples (S3 Fig), and a soft-thresholding power of β = 5 identified 10 modules (Fig 2D and 2E), with the MEturquoise module showing the highest correlation with the tumor phenotype (r = 0.61, p = 6 × 10 ⁻ ⁴⁵) (Fig 2F). Finally, by intersecting the key modules from both datasets with the mitochondrial metabolism gene set, 10 candidate genes were obtained, including *HSPA1A*, *LPIN2*, *TMEM25*, *ADI1*, *ANXA4*, *NCAPD2*, *PM20D2*, *SPSB2*, *FOXP4*, and *ELOVL5* (Fig 2G).

### 3.3. Intersection genes and functional annotation

**Fig 3:**
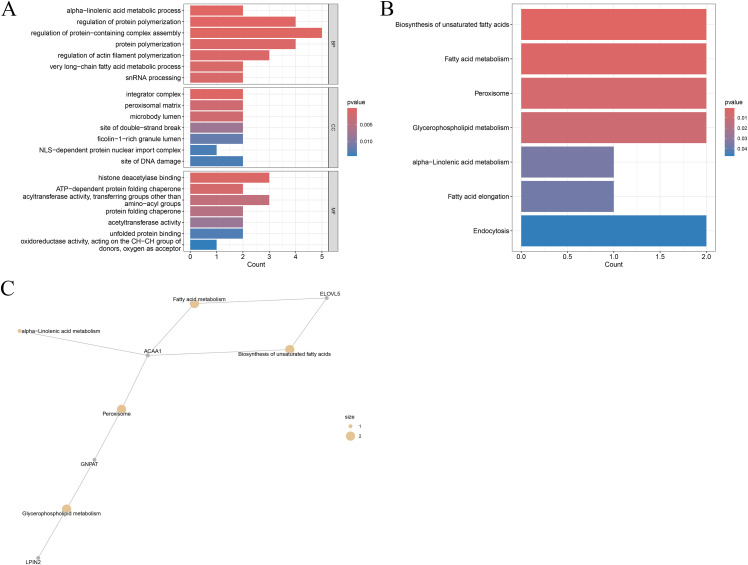
Functional Enrichment of Core Genes. A. GO functional annotation analysis of core genes, categorized into Biological Process (BP), Cellular Component (CC), and Molecular Function (MF). B. KEGG pathway enrichment analysis of core genes. C. Concept network plot of core genes and their associated KEGG pathways.

Merging DEGs with genes from significant WGCNA modules and removing duplicates resulted in 27 core genes. GO enrichment revealed strong involvement in fatty acid metabolism (e.g., very long-chain fatty acid metabolic process), epigenetic regulation (histone deacetylase binding), and cellular stress responses (DNA damage repair localization) (Fig 3A). KEGG analysis highlighted roles in lipid metabolic remodeling, including fatty acid metabolism, peroxisome, glycerophospholipid metabolism, and endocytosis pathways (p < 0.05, Fig 3B). Concept network analysis identified *ACAA1* as a central node linking peroxisome and fatty acid metabolism pathways (Fig 3C). These 27 core genes likely contribute to molecular mechanisms linking obesity and liver cancer through coordinated regulation of lipid metabolism, epigenetic modification, and subcellular organelle function.

### 3.4. ML-based selection and external validation

**Fig 4:**
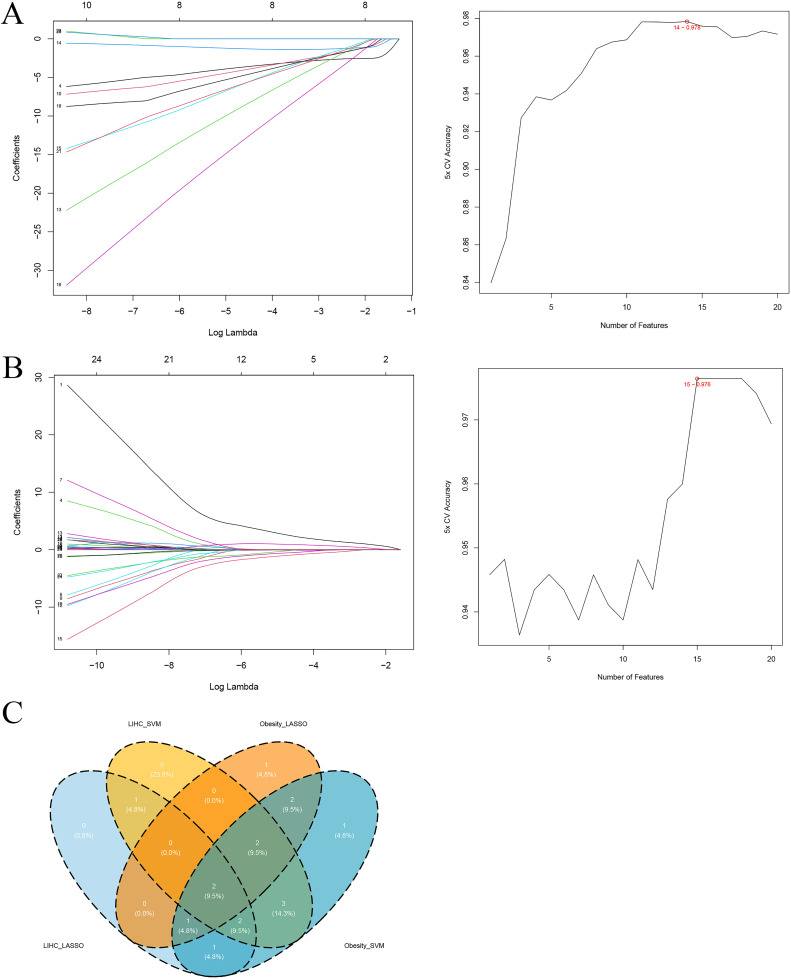
ML-Based Hub Gene Selection. A. Candidate genes identified by LASSO regression and SVM-RFE in the obesity dataset. B. Candidate genes identified by LASSO regression and SVM-RFE in the HCC dataset. C. Venn diagram depicting overlapping genes identified by both algorithms in the two disease datasets.

LASSO regression and SVM-RFE algorithms screened hub genes. In the obesity dataset, LASSO and SVM-RFE identified 8 and 14 candidate genes; integration produced 7 feature genes: *ARPC5*, *HDAC4*, *ACAA1*, *OFD1*, *SPRYD4*, *TMOD4*, and *ADI1* (Fig 4A). In the HCC dataset, 7 and 15 candidate genes were identified by LASSO regression and SVM-RFE (Fig 4B). Combined screening resulted in 5 feature genes: *MSTO1*, *ACAA1*, *LPIN2*, *ADI1*, and *SPSB2*. Venn diagram analysis revealed two overlapping genes between the two disease datasets: *ACAA1* and *ADI1* (Fig 4C). DEA confirmed that both *ACAA1* and *ADI1* were significantly downregulated in the obesity and HCC groups compared with their respective controls.

**Fig 5:**
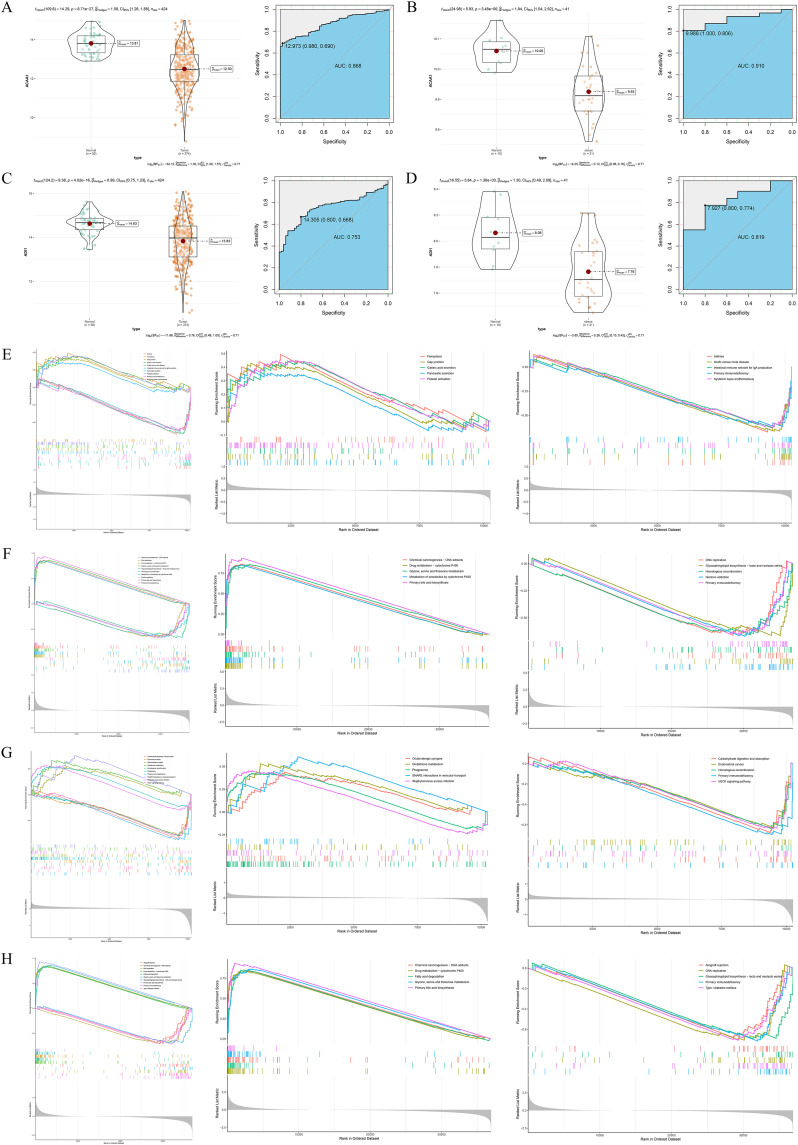
Identification and Functional Characterization of Diagnostic Genes in the Training Cohorts. A. DEA and ROC analysis of *ACAA1* in the HCC training dataset. B. DEA and ROC analysis of *ACAA1* in the obesity training dataset. C. DEA and ROC analysis of *ADI1* in the HCC training dataset. D. DEA and ROC analysis of *ADI1* in the obesity training dataset. E. GSEA of *ADI1* in the obesity cohort, including enrichment curves based on genome-wide gene ranking, and upregulated and downregulated pathway analyses. F. GSEA of *ADI1* in the HCC cohort, including enrichment curves based on genome-wide gene ranking, and upregulated and downregulated pathway analyses. G. GSEA of *ACAA1* in the obesity cohort, including enrichment curves based on genome-wide gene ranking, and upregulated and downregulated pathway analyses. H. GSEA of *ACAA1* in the HCC cohort, including enrichment curves based on genome-wide gene ranking, and upregulated and downregulated pathway analyses.

ROC curve analysis demonstrated robust discriminative capacity: *ACAA1* achieved AUCs of 0.868 and 0.910 in HCC and obesity cohorts (Fig 5A and 5B), while *ADI1* showed AUCs of 0.753 and 0.819 (Fig 5C and 5D). GSEA revealed *ADI1* was negatively correlated with immune dysregulation pathways, such as primary immunodeficiency in obesity (Fig 5E) and DNA replication/homologous recombination in HCC (Fig 5F). *ACAA1* was implicated in glutathione metabolism, fatty acid degradation, and primary bile acid biosynthesis (Fig 5G and 5H). Therefore, it is central for both genes in lipid metabolic remodeling and immune regulation.

**Fig 6:**
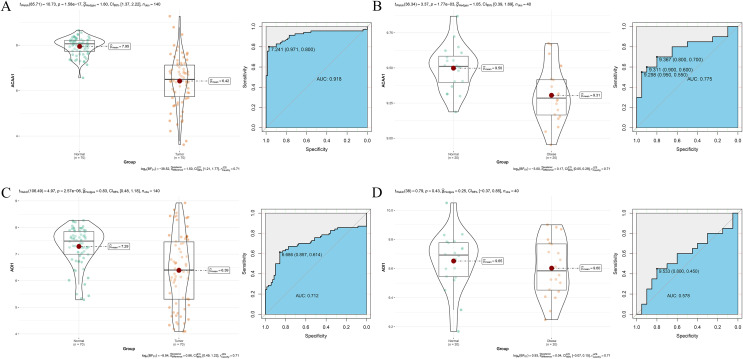
Performance Validation of Candidate Genes in External Cohorts. A. DEA and ROC analysis of *ACAA1* in the HCC validation dataset. B. DEA and ROC analysis of *ACAA1* in the obesity validation dataset. C. DEA and ROC analysis of *ADI1* in the HCC validation dataset. D. DEA and ROC analysis of *ADI1* in the obesity validation dataset.

In the external validation, *ACAA1* demonstrated robust diagnostic performance in both the HCC dataset GSE144269 (AUC = 0.918, Fig 6A) and the obesity dataset GSE151839 (AUC = 0.775, Fig 6B). In contrast, *ADI1* showed variable predictive performance, with an AUC of 0.712 in the HCC dataset (Fig 6C) and a relatively lower AUC of 0.578 in the obesity cohort (Fig 6D), suggesting its robustness as a standalone biomarker may be comparatively limited in the context of general obesity.

### 3.5. Correlation analysis of immune cell infiltration

**Fig 7:**
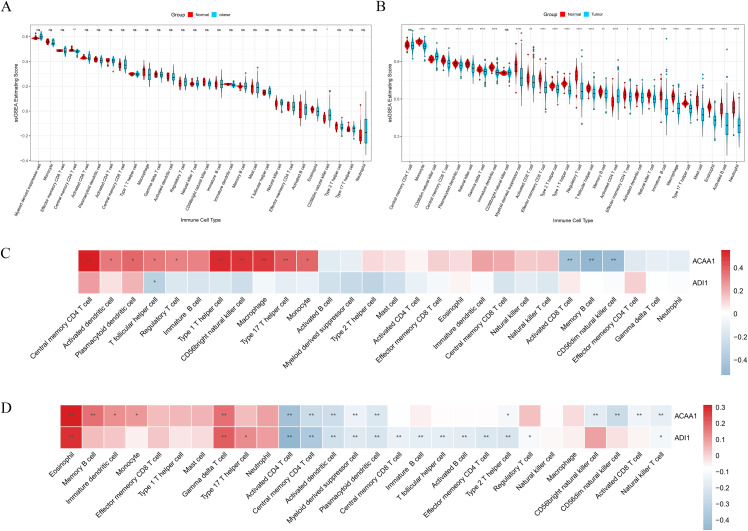
Immune Infiltration Analysis. A. Differences in immune cell distribution in the obesity dataset by ssGSEA. B. Differences in immune cell distribution in the HCC dataset by ssGSEA. C. Correlation between diagnostic genes and immune cells in the obesity dataset. The color bar represents the correlation coefficient (r). D. Correlation between diagnostic genes and immune cells in the HCC dataset. The color bar represents the correlation coefficient (r). Significance levels are indicated as follows: * P < 0.05, ** P < 0.01.

ssGSEA revealed distinct immune landscapes. In obesity, changes were limited to a moderate decrease in central memory CD4 T cells and a slight increase in CD56dim NK cells (Fig 7A). HCC showed extensive immune reprogramming, with significant enrichment of myeloid-derived suppressor cells (MDSCs) and Tregs, along with increased infiltration of activated and effective memory CD8 T cells (Fig 7B).

Correlation analysis indicated that in the obesity cohort (Fig 7C), *ACAA1* was positively correlated with multiple immune cells, including strong correlations with central memory CD4 T cells (r = 0.55), Type 1 T helper cells (r = 0.52), and macrophages (r = 0.49), as well as regulatory T cells (r = 0.31). Conversely, *ACAA1* exhibited moderate negative correlations with activated CD8 T cells (r=−0.44), memory B cells (r=−0.53), and CD56^dim^ NK cells (r=−0.48). *ADI1* showed a moderate negative correlation with T follicular helper cells (r=−0.33).

In the HCC cohort (Fig 7D), *ACAA1* was moderately positively correlated with eosinophils (r = 0.31), but only weakly with memory B cells (r = 0.17) and gamma delta T cells (r = 0.17); meanwhile, it was moderately negatively correlated with activated CD4 T cells (r=−0.39), central memory CD4 T cells (r=−0.26), and activated dendritic cells (r=−0.25). *ADI1* exhibited a similar overall pattern to *ACAA1*: positive correlations with eosinophils (r = 0.28) and gamma delta T cells (r = 0.20), and moderate-to-strong negative correlations with activated CD4 T cells (r=−0.46), central memory CD4 T cells (r=−0.36), and activated dendritic cells (r=−0.23), as well as a weak negative correlation with MDSCs (r=−0.18).

### 3.6. Correlation analysis of immune checkpoints and chemokines

**Fig 8:**
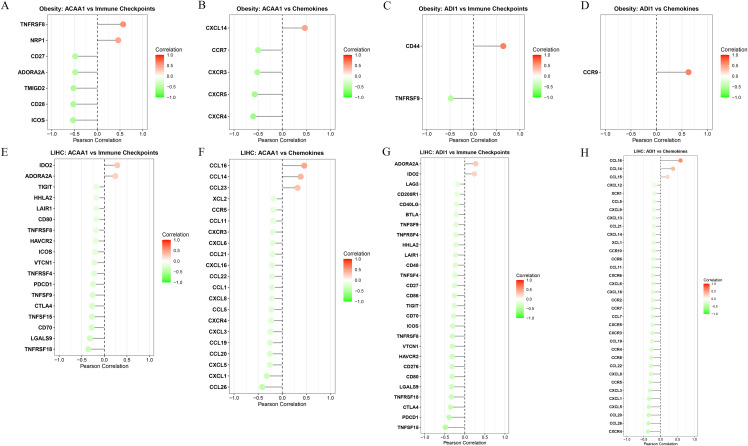
Immune-related Analyses of *ACAA1* and *ADI1* in Obesity and HCC Cohorts. A. Correlation between *ACAA1* and immune checkpoints in obesity. B. Correlation between *ACAA1* and chemokines in obesity. C. Correlation between *ADI1* and immune checkpoints in obesity. D. Correlation between *ADI1* and chemokines in obesity. E. Correlation between *ACAA1* and immune checkpoints in HCC. F. Correlation between *ACAA1* and chemokines in HCC. G. Correlation between *ADI1* and immune checkpoints in HCC. H. Correlation between *ADI1* and chemokines in HCC.

In the obesity cohort, *ACAA1* was positively correlated with immune checkpoint genes *TNFRSF8* (r = 0.57) and *NRP1* (r = 0.46), but moderately negatively correlated with canonical immune co-stimulatory molecules *ICOS* (r=−0.53), *CD27* (r=−0.48), *CD28* (r=−0.53), *TMIGD2* (r=−0.52), and *ADORA2A* (r=−0.49) (Fig 8A). Regarding chemokines, *ACAA1* showed significant-to-moderate negative correlations with immune cell migration receptors *CXCR4* (r=−0.60), *CXCR5* (r=−0.57), *CXCR3* (r=−0.52), and *CCR7* (r=−0.50), whereas only *CXCL14* (r = 0.47) exhibited a moderate positive correlation (Fig 8B). *ADI1* displayed more limited correlations, primarily showing significant positive correlations with *CD44* (r = 0.64) and *CCR9* (r = 0.63), and a moderate negative correlation with *TNFRSF9* (r=−0.48) (Fig 8C and 8D).

In the HCC cohort, *ACAA1* was negatively correlated with multiple immune checkpoint molecules, including PDCD1 (r=−0.25), *CTLA4* (r=−0.27), *CD70* (r=−0.28), and *TNFSF15* (r=−0.28), with stronger negative correlations observed for *TNFRSF18* (r=−0.36) and *LGALS9* (r=−0.32) (Fig 8E). Regarding chemokines, *ACAA1* showed positive correlations with *CCL14* (r = 0.38) and *CCL16* (r = 0.46) (Fig 8F). In contrast, *ADI1* exhibited more pronounced negative correlations, particularly with *PDCD1* (r=−0.40) and *TNFSF15* (r=−0.49), as well as *CTLA4* (r=−0.36), *LGALS9* (r=−0.33), *TNFRSF18* (r=−0.34), and *CD80* (r=−0.32) (Fig 8G), while showing consistent negative correlations with multiple chemokines, including *CXCR4* (r=−0.37), *CCL20* (r=−0.36), *CXCL5* (r=−0.35), *CXCL1* (r=−0.34), *CXCL3* (r=−0.32), *CCL26* (r=−0.36), and *CCR5* (r=−0.30) (Fig 8H).

## 4. Discussion

This study was based on the hypothesis that mitochondrial metabolic dysregulation mediates obesity-driven HCC. By integrating transcriptomic signatures through DEGs, WGCNA, and ML, *ACAA1* and *ADI1* were identified as consistently downregulated candidate genes across both disease contexts. Despite the inherent tissue discordance, obesity data from PBMCs and HCC data from liver tissue, the consistent expression patterns of these genes highlight their potential as systemic metabolic sensors, suggesting that blood-based expression changes may reflect early metabolic reprogramming preceding hepatic pathology. *ACAA1* demonstrated robust diagnostic performance across cohorts, whereas *ADI1* showed more variable results in obesity external validation, functioning as a key regulatory candidate gene within the metabolic-immune axis rather than a standalone biomarker. Moreover, immune infiltration analyses further implied significant correlations of both genes with multiple immune cell subsets, supporting their potential role in remodeling the tumor microenvironment via lipid metabolism and immune homeostasis regulation.

Functional annotation indicated predominant enrichment of these genes in lipid metabolic pathways, including fatty acid degradation, unsaturated fatty acid biosynthesis, and bile acid metabolism, all closely linked to mitochondrial function. In obesity, hepatocyte accumulation of saturated fatty acids coupled with impaired β-oxidation generates excessive reactive oxygen species (ROS) and toxic lipid byproducts, causing mitochondrial damage and cellular injury, thereby providing a metabolic basis for hepatocarcinogenesis [30]. SREBP transcription factors are also activated in obesity, promoting monounsaturated fatty acid synthesis to partially alleviate lipotoxicity; however, overaccumulation of SCD1-mediated products exacerbates hepatic steatosis and mitochondrial stress, potentially facilitating HCC development [31,32]. Obesity-associated gut microbiota dysbiosis can lead to secondary bile acid accumulation, which disrupts mitochondrial membranes, inhibits the respiratory chain, and activates oncogenic pathways such as mTOR, promoting the transition from MASH to HCC [33].

*ACAA1* serves as a terminal enzyme in peroxisomal fatty acid β-oxidation [34] and its clinical relevance in liver cancer is underscored by its consistent downregulation in HCC tissues and aggressive cell lines [35], and its predictive value for patient survival and immune cell infiltration [36]. Our mitochondria-focused analyses suggest that in obesity, *ACAA1* deficiency creates a lipotoxic cascade bridging peroxisomal dysfunction with mitochondrial failure. Acetyl-CoA, NADH, and incompletely oxidized short-chain fatty acids produced by peroxisomal β-oxidation are typically shuttled to mitochondria for complete oxidation [37,38], making peroxisomal integrity essential for mitochondrial metabolic homeostasis. This metabolic synergy is particularly vulnerable under obesity-associated hepatic lipid overload, potentially exacerbated by dysregulated endocytic uptake [39]. *ACAA1* downregulation may impair peroxisomal clearance of mitochondria-challenging substrates, such as very-long-chain fatty acids (VLCFAs), leading to lipid intermediate accumulation and the generation of reactive byproducts like H₂O₂ [40,41]. Oxidative stress could be further amplified by deficient antioxidant defenses, as suggested by GSEA findings linking *ACAA1* expression with glutathione metabolism in obesity [42]. These metabolites may inflict secondary mitochondrial damage, compromising membrane integrity and electron transport chain efficiency [43]. Beyond its catabolic role, *ACAA1* deficiency may hinder hepatocellular adaptation by interfering with the synthesis of peroxisome-derived ether lipids, which modulate mitochondrial dynamics, including fission-fusion balance, and are essential for assembling respiratory supercomplexes [44,45]. Depletion of these lipids under obesity-related stress could further destabilize mitochondrial architecture, rendering hepatocytes more susceptible to malignant transformation. Therefore, *ACAA1* downregulation potentially converts chronic obesity-related metabolic pressure into a peroxisome-to-mitochondria lipotoxic cascade, contributing to MAFLD progression toward HCC.

In contrast, the role of *ADI1* in obesity and liver cancer remains incompletely defined. *ADI1* encodes a metal-binding enzyme critical for the methionine salvage pathway [46,47] and exhibits tumor suppressive activity in HCC through modulation of S-adenosylmethionine (SAMe) levels and DNA methylation [48]. *ADI1* showed stronger diagnostic performance in HCC validation cohort than in external obesity cohorts. This discrepancy suggests that the biological relevance of *ADI1* may be more specifically tied to the oncogenic process and metabolic-immune reprogramming rather than serving as a generalized biomarker for obesity. Building on its role in SAMe metabolism, we hypothesize that *ADI1* downregulation may contribute to multifaceted metabolic disruption in the obese liver. SAMe depletion could limit glutathione synthesis and compromise mitochondrial membrane integrity via reduced phosphatidylcholine and coenzyme Q10 production [49–52]. Furthermore, as the primary methyl donor for DNA, RNA, and histone methylation, SAMe depletion is associated with global hypomethylation, a well-established driver of malignant progression. While these mechanistic interpretations remain to be experimentally validated, GSEA results link reduced *ADI1* expression to pathways associated with genomic instability [53–55]. Therefore, *ADI1* downregulation may transform obesity-induced metabolic stress into mitochondrial dysfunction and epigenetic dysregulation, facilitating the progression from fatty liver disease to HCC.

To investigate how the downregulation of *ACAA1* and *ADI1* relates to metabolic dysregulation with the pro-tumorigenic TME, ssGSEA infiltration scores with correlation analyses were performed. The generally modest correlation coefficients (r = 0.2–0.4) between these hub genes and immune parameters likely reflect the highly complex, multifactorial nature of the TME, where single metabolic genes rarely exert dominant control over specific immune subsets. Rather than statistical noise, the consistent trends across large datasets suggest that *ACAA1* and *ADI1* are contributing components within a broader metabolic-immune regulatory network. Notably, the immune landscape displays stage-specific evolution from obesity to HCC, characterized by sign reversals in correlation patterns. In obesity, *ACAA1* expression positively correlates with central memory CD4 ⁺ T cells and Th1 cells, indicating that early downregulation may impair protective immune memory and helper function. Conversely, negative correlations with activated CD8 ⁺ T cells and CD56^dim^ NK cells in obesity suggest an initial compensatory inflammatory recruitment of effector cells in response to metabolic stress.

However, as the disease progresses to HCC, the immune pattern shifts toward a predominantly immunosuppressive state. Although *ACAA1* and *ADI1* continue to show negative correlations with activated T cells and NK cells, indicating ongoing effector recruitment, ssGSEA scores reveal significant enrichment of MDSCs and Tregs. This reflects a transition to “signal amplification without effective response,” in which effector recruitment is counteracted by an inhibitory microenvironment. The root potentially lies in the remodeling effect of the obesity-related lipid microenvironment on immune cells: lipid accumulation systematically impairs the effector potential of anti-tumor immune cells [56,57]. In CD8 ⁺ T cells, lipid overload is associated with the upregulation of *PD-1* and *CTLA-4* alongside the downregulation of cytotoxic markers such as *GZMB* and *IFN-γ*, where oxidative stress further exacerbates functional decline [58–61]. Despite being recruited, NK cells may exhibit a “pseudo-activated” CD69 ⁺ phenotype but show impaired cytotoxicity and a shift toward a low-cytotoxic ILC1-like state in hepatic tissue [62]. In contrast, MDSCs and Tregs can sustain and enhance their immunosuppressive functions via increased fatty acid oxidation [63,64].

Building on this metabolic foundation, lipid-driven immune remodeling is further mediated by dysregulated signaling networks downstream of candidate gene deficiency. First, the downregulation of *ACAA1* and *ADI1* is associated with upregulation of multiple inhibitory pathways, including *PD-1*, *CTLA-4*, and *LGALS9*. This synergy may further drive effector T cells into irreversible functional exhaustion, especially as these cells are already compromised by the lipid environment [65,66]. Such exhaustion-like gene expression profiles align with observations in obese murine models where adipose-resident T cells display significant functional decline [67]. Second, *ACAA1* and *ADI1* downregulation is accompanied by enhanced axes like *CXCR4*, *CCL20*, *CXCL1/3/5*, and *CCR5*, indicating chemokine network dysregulation that potentially leads to ineffective immune cell recruitment and physical sequestration. Specifically, the *CXCR4-CXCL12* axis may sequester T cells within the tumor stroma. The upregulation of *CXCL1/3/5* and *CCR5* likely promotes the recruitment of immunosuppressive populations, notably MDSCs, into the tumor core [68,69]. This phenomenon mirrors the clinical landscape in MASH patients, in which accumulation of CD8 ⁺ PD-1 ⁺ exhausted T cells within the liver has been reported to impede immunotherapeutic efficacy [70]. Our findings suggest that the coordinated deficiency of *ACAA1* and *ADI1* is associated with patterns of effector cell “functional exhaustion” and “spatial segregation,” shaping the immunosuppressive microenvironment of obesity-associated HCC.

In summary, this study proposes a novel theoretical framework centered on the obesity-mitochondria-immune axis, providing evidence that *ACAA1* and potentially *ADI1* may act as metabolic-immunoregulatory bridges linking obesity to HCC. Beyond classical metabolic signatures focused on glycolysis or lipogenesis, our work identifies these genes as candidates potentially involved in peroxisomal-mitochondrial crosstalk and methionine homeostasis, respectively. Leveraging multiomics datasets and ML, the findings offer a previously underexplored theoretical hypothesis for how obesity-associated metabolic stress contributes to a pro-tumor immunosuppressive microenvironment. The novelty of this work lies in delineating this mechanistic axis, which remains underexplored in current hepatocarcinogenesis research.

However, these results are bioinformatically derived hypotheses rather than definitive mechanistic proof. Key limitations include tissue discordance, with obesity signatures from PBMCs and HCC data from liver tissue, compounded by the use of skin and fat tissue in obesity external validation. Suboptimal diagnostic performance of *ADI1* in the obesity validation cohort (AUC = 0.578) further suggests its role as a functional component within the metabolic-immune axis rather than a standalone biomarker. Variability may also reflect relatively small obesity cohort sizes and discrepancies in clinical characteristics, such as the mismatched sex distribution and age ranges between training and validation cohorts. As the analysis relies on public databases, findings require independent validation at the protein level and in larger, prospective clinical cohorts.

Future studies should focus on larger, demographically balanced (matched for age and sex) and liver-specific obesity cohorts while integrating cellular or animal experiments to translate these bioinformatic associations into mechanistic proof. Elucidating the functional roles of *ACAA1* and *ADI1* will clarify their translational potential in obesity-related HCC and provide a theoretical basis for precision interventions targeting the metabolic-immune axis.

## 5. Conclusion

This study proposes a novel theoretical framework centered on the obesity-mitochondria-immune axis. Through DEA, WGCNA, and ML algorithms, *ACAA1* and potentially *ADI1* were identified as candidate genes consistently downregulated in both obesity and HCC. *ACAA1* downregulation may impair mitochondrial function through peroxisome–mitochondria lipotoxic crosstalk, while *ADI1* downregulation may deplete the key metabolite SAMe, collectively compromising epigenetic stability and mitochondrial integrity. These alterations appear to drive the development of an immunosuppressive tumor microenvironment. As the study lacks direct experimental verification, these findings should be considered hypotheses pending protein-level and prospective clinical validation. Overall, the results provide preliminary insights into potential candidate genes and the metabolic–immune reprogramming underlying HCC progression.

## Supporting information

S1 Table Detailed clinical characteristics and metadata of the included datasets. (DOCX)

S1 Fig Evaluation of batch effect removal in merged obesity datasets. (TIF)

S2 Fig Sample clustering and outlier detection for the obesity dataset. (TIF)

S3 Fig Sample clustering and outlier detection for the HCC dataset. (TIF)
