# Supplementary material for: Identification of core genes mediating the association between obesity and hepatocellular carcinoma: A bioinformatics study based on mitochondrial metabolism and immune pathways
Source: PLoS One. 2026 Mar 9;21(3):e0344452. doi: 10.1371/journal.pone.0344452 (PMC12970922; doi:10.1371/journal.pone.0344452)
Supplement: S1 Table — (DOCX) [file pone.0344452.s001.docx]

**S1 Table.** Detailed Clinical Characteristics and Metadata of the Included Datasets.

| Category | Phenotype | Dataset | Platform | Tissue Type | Sample Size (Cases/Controls) | Median  (Age Range) | Sex (M/F) | Obesity Criteria  (BMI) |
| --- | --- | --- | --- | --- | --- | --- | --- | --- |
| Training Phase | HCC | TCGA-LIHC | ENSEMBL | Liver tissue | 374 / 50 | 62  (16–90) | 281/143 | \ |
| Training Phase | Obesity | GSE55205 | GPL10558 | PBMC | 17 / 6 | 40  (20-58) | 23 / 0 | ≥ 25 kg/m² |
| Training Phase | Obesity | GSE69039 | GPL10558 | PBMC | 14 / 4 | (20-59) | 18 / 0 | ≥ 25 kg/m² |
| Validation Phase | Obesity | GSE151839 | GPL570 | Skin and fat | 20 / 20 | (48-69) | 0 / 40 | 35-50 kg/m² |
| Validation Phase | HCC | GSE144269 | GPL24676 | Liver tissue | 70 / 70 | 60 | 37 / 38 | \ |

All data analyzed in this study were obtained from publicly available databases (TCGA and GEO).
