# Supplementary figures and images for: Identification of core genes mediating the association between obesity and hepatocellular carcinoma: A bioinformatics study based on mitochondrial metabolism and immune pathways
Source: PLoS One. 2026 Mar 9;21(3):e0344452. doi: 10.1371/journal.pone.0344452 (PMC12970922; doi:10.1371/journal.pone.0344452)

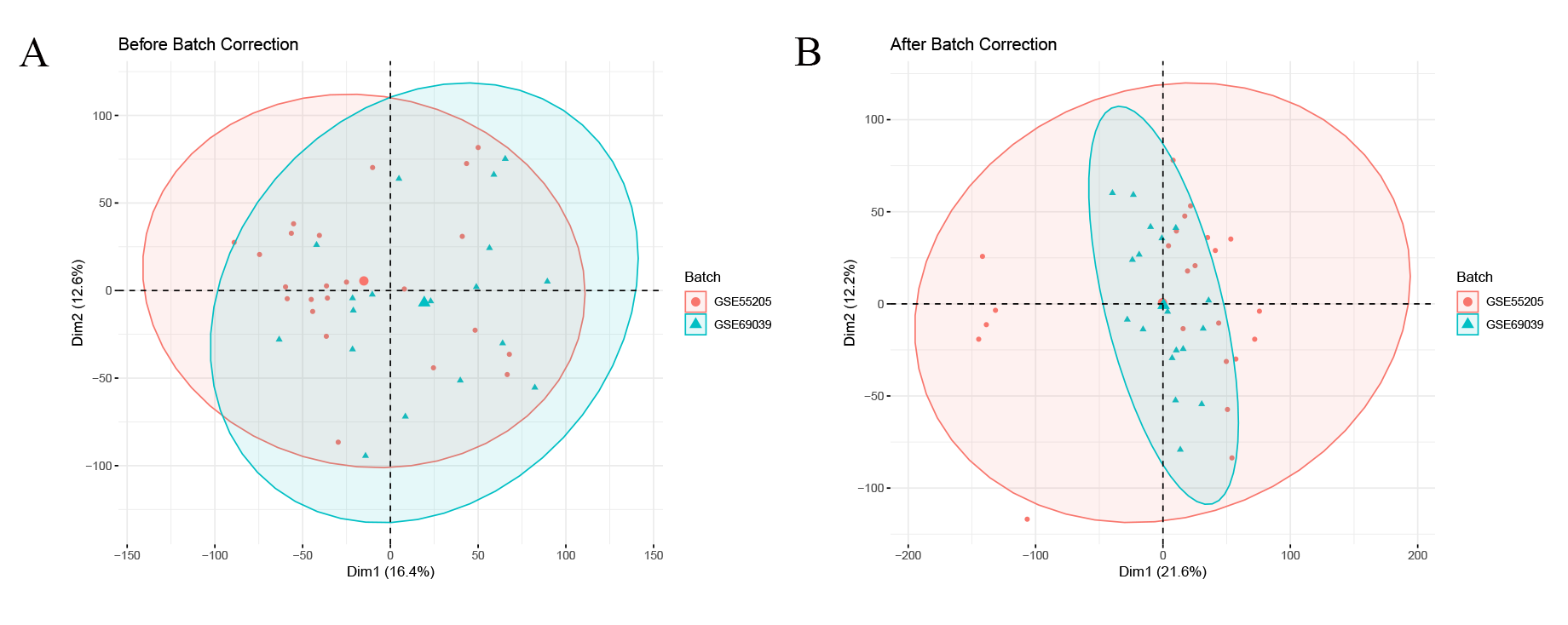

Supplement: S1 Fig — (TIF) [file pone.0344452.s002.tif]

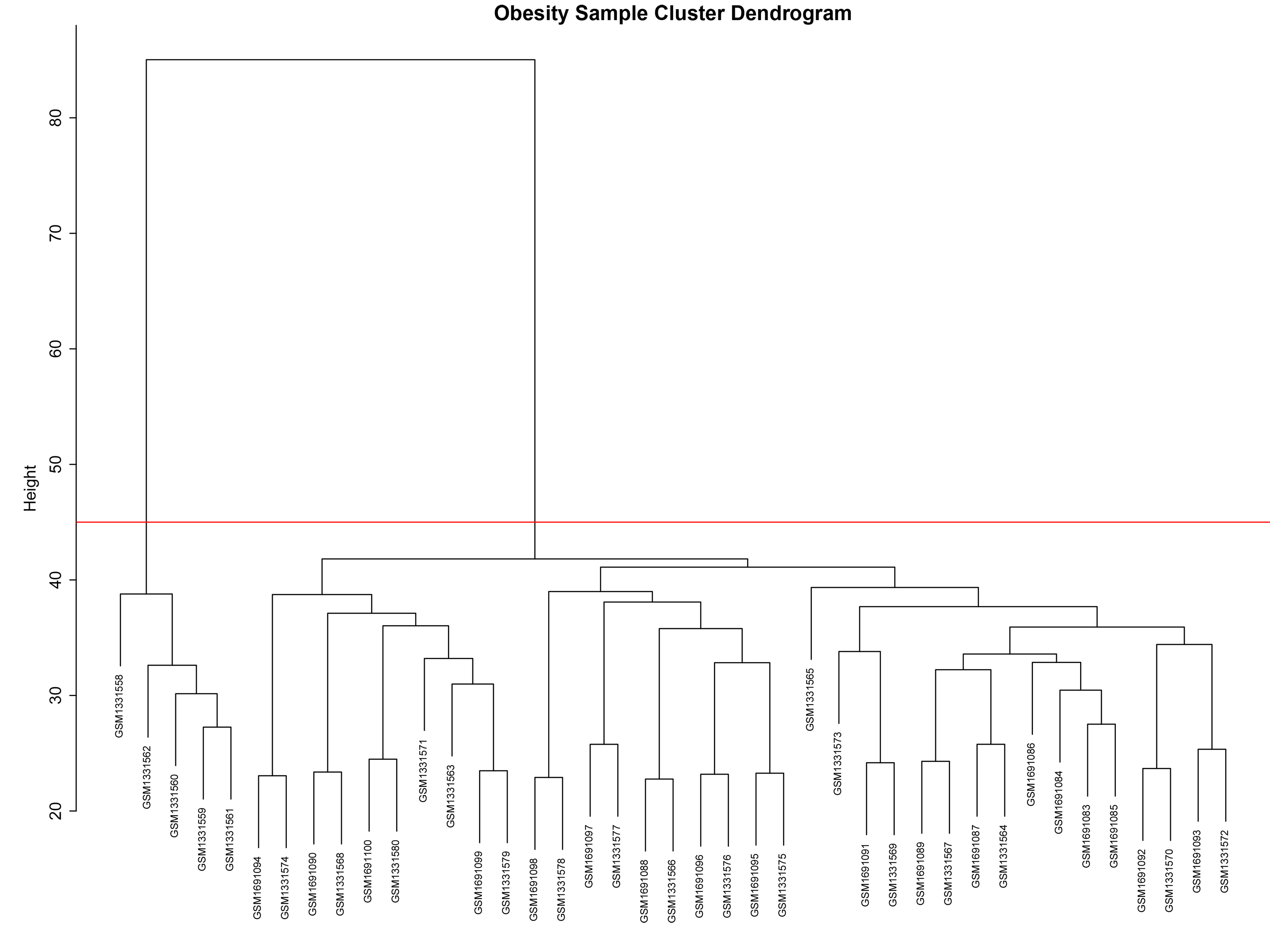

Supplement: S2 Fig — (TIF) [file pone.0344452.s003.tif]

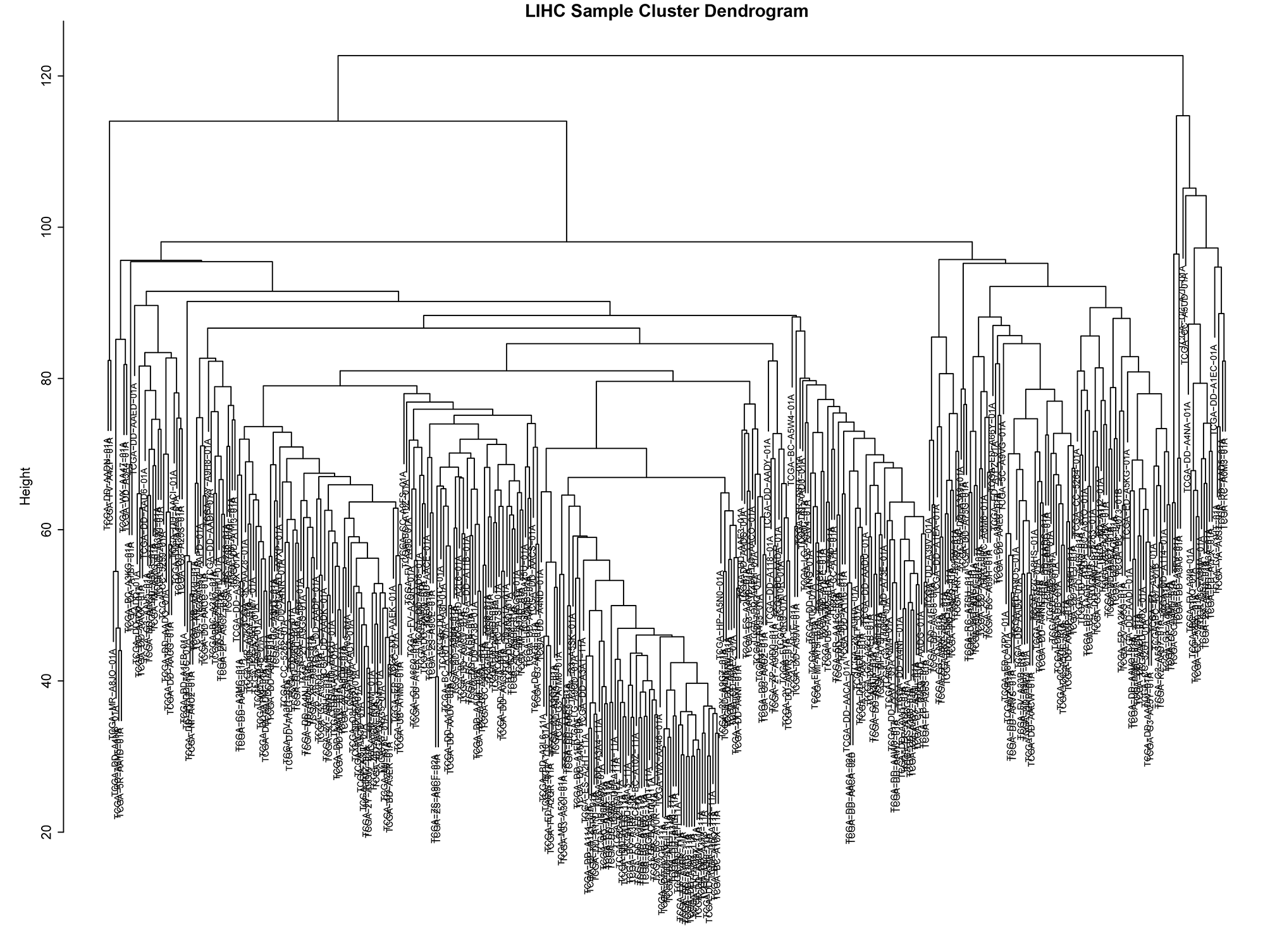

Supplement: S3 Fig — (TIF) [file pone.0344452.s004.tif]
